# Supplementary material for: Sensitization of Cutaneous Primary Afferents in Bone Cancer Revealed by In Vivo Calcium Imaging
Source: Cancers (Basel). 2020 Nov 24;12(12):3491. doi: 10.3390/cancers12123491 (PMC7760605; doi:10.3390/cancers12123491)
Supplement: Supplementary file 1 [file cancers-12-03491-s001.zip › cancers-989779-Supplementary/cancers-989779-supplementary-update.pdf]

Sensitization of Cutaneous Primary Afferents in Bone Cancer Revealed by in vivo Calcium Imaging

Larissa de Clauser, Ana P. Luiz, Sonia Santana-Varela, John N. Wood and Shafaq Sikandar

Table S1. Primers used for genotyping.

PCR Product	Forward primer	Reverse Primer
	**Rosa-flox-stop tdTomato (Ai9)**	
WT (297 b.p.)	AAGGGAGCTGCAGTGGAGTA	CCGAAAATCTGTGGGAAGTC
tdTomato (196 b.p.)	CTGTTCCTGTACGGCATGG	GGCATTAAAGCAGCGTATCC
	**Scn10a-Cre**	
Scn10a WT (258 b.p.)	CAGTGGTCAGGCTGTCACCA	ACAGGCCTTCAAGTCCAACTG
Cre (346 b.p.)	CAGTGGTCAGGCTGTCACCA	AAATGTTGCTGGATAGTTTTTACTGCC
	**Tmem233-Cre**	
Tmem233 WT (361 b.p.)	TCATCTCCCTTGAGCCCGGAG	GAGTCTGAGCGGGAAGCATACT
Cre (154 b.p.)	CCTGCTGTCCATTCCTTATTCCAT	GAGTCTGAGCGGGAAGCATACT
	**Pvalb-cre**	
Pvalb WT (500 b.p.)	CAGAGCAGGCATGGTGACTA	AGTACCAAGCAGGCAGGAGA
Cre (100 b.p.)	GCGGTCTGGCAGTAAAAACTATC	GTGAAACAGCATTGCTGTCACTT
	**Calb1-Cre**	
Calb1 WT (311 b.p.)	AGAACATAATGGCCTTGTCG	TACTGACTGGCCTAAGCATGG
Cre (144 b.p.)	AGAACATAATGGCCTTGTCG	ACACCGGCCTTATTCCAAG
	**Pirt-GCaMP3**	
WT (404 b.p.)	TCCCCTCTACTGAGAGCCAG	GGCCCTATCATCCTGAGCAC
Pirt-GCaMP3 (300 b.p.)	TCCCCTCTACTGAGAGCCAG	ATAGCTCTGACTGCGTGACC

Table S2. Overview of animals used for behavioural testing.

**Experiment**	**Figure**	**Number of animals**	**Strain**
Limb use score, weight bearing	Figure 2A-B	Sham: *n* = 22<br>Cancer: *n* =17	Sham: Pirt-GCaMP3 (*n* = 11), Pirt-GCaMP3/Calb1-cretdTomato (*n* = 3), Pirt-GCaMP3/Tmem233-cretdTomato (*n* = 8)<br>Cancer: Pirt-GCaMP3 (*n* = 12), Pirt-GCaMP3/Calb1-cretdTomato (*n* = 1), Pirt-GCaMP3/Tmem233-cretdTomato (*n* = 4)
von Frey	Figure 2C	Sham: *n* = 21<br>Cancer: *n* =12	Same as Limb use score and weight bearing, but excluding the following animals as behavioural testing at the endpoint was not possible:<br>Cancer: Pirt-GCaMP3 (*n* = 4), Pirt-GCaMP3/Tmem233-cretdTomato (*n* = 1)<br>Sham: Pirt-GCaMP3 (*n* = 1)
Noxious palpation	Figure 2D	Sham: *n*=11<br>Cancer: *n* = 5	Sham: Pirt-GCaMP3/Calb1-cretdTomato (*n* = 3), Pirt-GCaMP3/Tmem233-cretdTomato (*n* = 8)<br>Cancer: Pirt-GCaMP3/Calb1-cretdTomato (*n* = 1), Pirt-GCaMP3/Tmem233-cretdTomato (*n* = 4)
Randall-Selitto	Figure 2E	Sham: *n* = 7<br>Cancer *n* = 6	Pirt-GCaMP3
Hot-plate	Figure 2F	Sham: *n* = 4<br>Cancer: *n* = 6	Pirt-GCaMP3

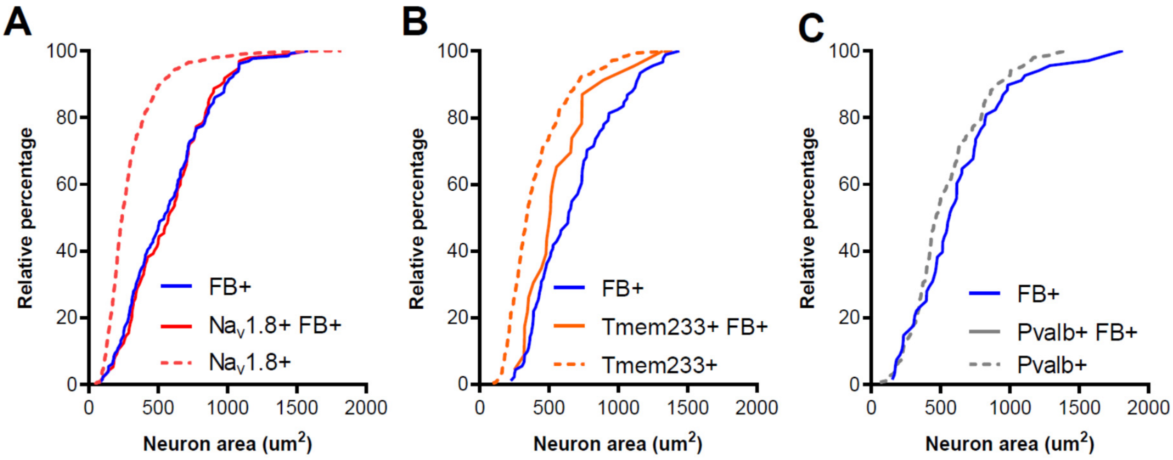

Figure S1. Size distribution of retrogradely labelled femoral bone marrow afferents within each genetically labelled neuronal subset; Supplementary methods including.

© 2020 by the authors. Licensee MDPI, Basel, Switzerland. This article is an open access article distributed under the terms and conditions of the Creative Commons Attribution (CC BY) license (http://creativecommons.org/licenses/by/4.0/).
